# Radiomic Features and Machine Learning for the Discrimination of Renal Tumor Histological Subtypes: A Pragmatic Study Using Clinical-Routine Computed Tomography

**DOI:** 10.3390/cancers12103010

**Published:** 2020-10-16

**Authors:** Johannes Uhlig, Andreas Leha, Laura M. Delonge, Anna-Maria Haack, Brian Shuch, Hyun S. Kim, Felix Bremmer, Lutz Trojan, Joachim Lotz, Annemarie Uhlig

**Affiliations:** 1 Department of Diagnostic and Interventional Radiology, University Medical Center Goettingen, Robert-Koch-Str. 40, 37075 Goettingen, Germany; johannes.uhlig@med.uni-goettingen.de (J.U.); lauramaria.delonge@stud.uni-goettingen.de (L.M.D.); annamaria.haack@stud.uni-goettingen.de (A.-M.H.); joachim.lotz@med.uni-goettingen.de (J.L.); 2 Department of Radiology and Biomedical Imaging, Division of Interventional Radiology, Yale School of Medicine, New Haven, CT 06510, USA; kevin.kim@yale.edu; 3 Department of Medical Statistics, University Medical Center Göttingen, Humboldtallee 32, 37073 Goettingen, Germany; andreas.leha@med.uni-goettingen.de; 4 Institute of Urologic Oncology, David Geffen School of Medicine at UCLA, Los Angeles, CA 90095, USA; bshush@mednet.ucla.edu; 5 Yale Cancer Center, Yale School of Medicine, New Haven, CT 06510, USA; 6 Institute of Pathology, University Medical Center Goettingen, Robert-Koch-Str. 40, 37075 Goettingen, Germany; felix.bremmer@med.uni-goettingen.de; 7 Department of Urology, University Medical Center Goettingen, Robert-Koch-Str. 40, 37075 Goettingen, Germany; lutz.trojan@med.uni-goettingen.de; 8 German Centre for Cardiovascular Research, Partnersite Goettingen, Robert-Koch-Str. 40, 37075 Goettingen, Germany

**Keywords:** renal cell carcinoma, computed tomography, image interpretation, computer-assisted, radiomics, machine learning

## Abstract

**Simple Summary:**

This study evaluates how advanced image analyses (radiomic features) and machine learning algorithms can help to distinguish subtypes of kidney tumors in computed tomography (CT) images, which is important for further patient treatment. For 201 patients, the image analyses showed a moderate performance, but robustly performed across various imaging centers and even in cases with suboptimal image quality. In particular, distinguishing one specific subtype of kidney tumor (oncocytomas) from other subtypes proves to be challenging. The algorithms presented in this study can help in the clinical decision-making process for kidney tumor patients, for example, to decide whether to perform kidney surgery or not.

**Abstract:**

This study evaluates the diagnostic performance of radiomic features and machine learning algorithms for renal tumor subtype assessment in venous computed tomography (CT) studies from clinical routine. Patients undergoing surgical resection and histopathological assessment of renal tumors at a tertiary referral center between 2012 and 2019 were included. Preoperative venous-phase CTs from multiple referring imaging centers were segmented, and standardized radiomic features extracted. After preprocessing, class imbalance handling, and feature selection, machine learning algorithms were used to predict renal tumor subtypes using 10-fold cross validation, assessed as multiclass area under the curve (AUC). In total, *n* = 201 patients were included (73.7% male; mean age 66 ± 11 years), with *n* = 131 clear cell renal cell carcinomas (ccRCC), *n* = 29 papillary RCC, *n* = 11 chromophobe RCC, *n* = 16 oncocytomas, and *n* = 14 angiomyolipomas (AML). An extreme gradient boosting algorithm demonstrated the highest accuracy (multiclass area under the curve (AUC) = 0.72). The worst discrimination was evident for oncocytomas vs. AML and oncocytomas vs. chromophobe RCC (AUC = 0.55 and AUC = 0.45, respectively). In sensitivity analyses excluding oncocytomas, a random forest algorithm showed the highest accuracy, with multiclass AUC = 0.78. Radiomic feature analyses from venous-phase CT acquired in clinical practice with subsequent machine learning can discriminate renal tumor subtypes with moderate accuracy. The classification of oncocytomas seems to be the most complex with the lowest accuracy.

## 1. Introduction

In 2018, renal cell carcinoma (RCC) accounted for approximately 175,000 cancer-related deaths worldwide [1]. Over the last decades, RCC incidence has been increasing at an annual rate of approximately 2%, which has been attributed to an earlier detection of small renal tumors through wider-spread use and technical advancements of cross-sectional imaging [2,3,4].

Depending on their cellular origin and behavior, renal tumors are classified into several histological subtypes with unique incidence, prognostic profile, and therapeutic options [5,6]. Despite the clinical implications of different renal tumor subtypes, their distinction by cross-sectional imaging remains imperfect: after surgical resection of renal tumors, approximately 20% of them are histopathologically assessed as benign, a percentage that increases up to 46.3% for renal tumors with a diameter <1 cm [7,8].

One promising approach to optimize radiological assessment is the utilization of radiomic feature analyses and machine learning (ML) algorithms, which have been shown to perform well for various classification problems and imaging modalities [9,10]. Although some studies on advanced image analyses in the context of renal tumors have been published, most were based on dedicated renal imaging protocols with standardized acquisition schemes [11,12,13]. To date, there is no evidence of how digital data exploitation can be applied for renal tumor subtype assessment in a real-life scenario where renal tumors are often incidentally detected on single venous-phase computed tomography (CT) studies acquired at different imaging centers with various protocols and varying quality.

This study thus aims to assess renal tumor subtypes with radiomic feature analyses and subsequent machine learning in a pragmatic approach utilizing CT studies from clinical routine.

## 2. Results

### 2.1. Study Cohort

**Figure 1:**
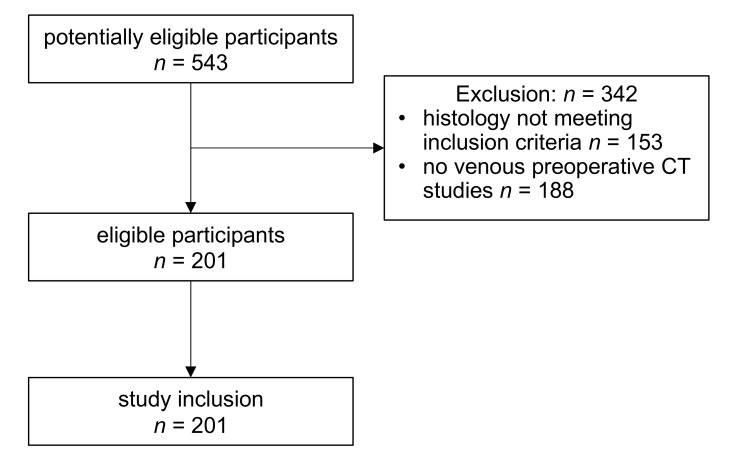
Flowchart of patient inclusion and exclusion.

This study’s cohort comprised *n* = 201 renal tumors from the same number of patients, of which *n* = 73 were female (36.3%), and *n* = 121 male (63.7%). The median age was 66 years (inter-quartile range (IQR): 56–74 years). Histopathological analyses revealed clear cell renal cell carcinomas (ccRCC) in *n* = 131 patients (70.8%), papillary RCC in *n* = 29 (15.7%), chromophobe RCC in *n* = 11 (5.9%), and angiomyolipomas (AML) in *n* = 14 (7.6%). A study flowchart is provided in Figure 1. The median of the largest three-dimensional (3D) diameter of the included renal tumors was 51.6 mm (IQR: 38.4–68.4 mm). The median CT slices’ thickness was 2 mm (IQR: 1–5 mm), with a total of *n* = 87 CT studies performed at a tertiary referral center (43.4%).

**Figure 2:**
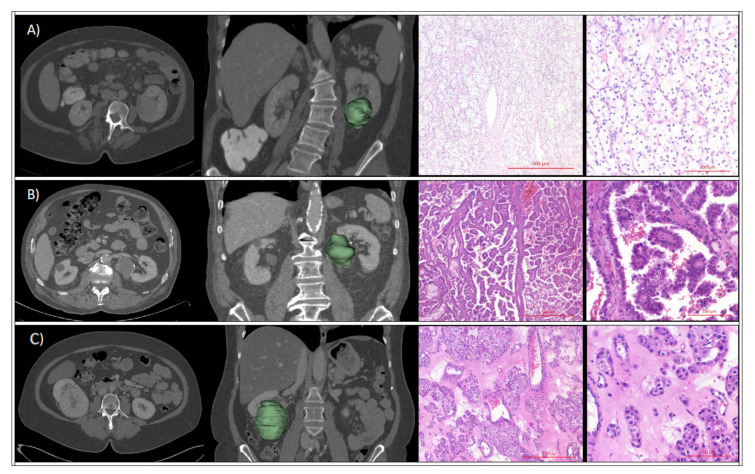
Clinical cases of patients presenting with: (**A**) left-sided renal tumor of the lower pole with heterogenous enhancement on venous-phase computed tomography (CT) with corresponding 3D-segmentation and histopathological HE-staining at 5 X and 20 X magnification, which revealed a ccRCC subtype; Scale Bar 500 µm and 100 µm; (**B**) left-sided central renal tumor with hypodense central attenuation, diagnosed as papillary RCC by histopathological analysis; Scale Bar 500 μm and 100 μm; (**C**) right-sided renal tumor of the middle and lower pole with heterogeneous enhancement and central scar-like hypodensity, that was confirmed as oncocytoma on histopathological assessment; Scale Bar 500 μm and 100 μm.

After stratification by renal tumor histology, no statistically significant differences were evident for the maximum 3D diameter (Table 1). Patients with AML (56 ± 14 years) or the chromophobe (60 ± 9.2 years) subtype were younger compared to patients with the papillary (67 ± 11 years) or ccRCC (65 ± 11 years) subtype (*p* = 0.02 for overall difference). While ccRCC and papillary RCC were more common in male patients, female patients showed a prevalence of AMLs and chromophobe RCC (64.3% vs. 35.7% and 63.6% vs. 36.4%, respectively; *p* < 0.01 for overall difference). Representative patient cases are provided in Figure 2. Additional information on histological grading and T stage of malignant cases is provided in Table S1.

As summarized in Table 2, imaging artifacts were observed in *n* = 60 CT studies (29.9%), the majority being motion artifacts (*n* = 56; 27.9%) and of moderate severity (*n* = 27; 13.4%). Metallic artifacts with severe image streaking, or combination of severe metallic and motion artifacts was evident in two cases each (1%, respectively).

### 2.2. Machine Learning Algorithms

To address data imbalance, the underrepresented subtypes (chromophobe and papillary RCC, AML, and oncocytoma) were augmented via synthetic upsampling to represent approximately 50% of the cases of the predominant renal tumor subtype ccRCC, using the synthetic minority oversampling technique (SMOTE). SMOTE generates new cases while preserving the attributes of the cohort. A summary of the patient characteristics in the SMOTE cohort is provided in Table 1.

Table 3 summarizes the diagnostic accuracy of different machine learning algorithms predicting the renal mass subtype in the full study cohort. Among the machine learning algorithms, extreme gradient boosting (xgboost) without feature selection but with upsampling achieved the numerically highest area under the curve (AUC) = 0.72. Across all machine learning models, feature selection resulted in diminishing performance for all but elastic-net penalized multinomial regression (mean AUC decrease 0.04). Feature extraction via principle component analysis (PCA) showed mixed effects: while the average effect over all machine learning models was negative (mean AUC decrease 0.026), the ranger implementation of random forests improved. Upsampling resulted in better classification performance both with feature selection (mean AUC increase 0.06) and without feature selection (mean AUC increase 0.04).

**Figure 3:**
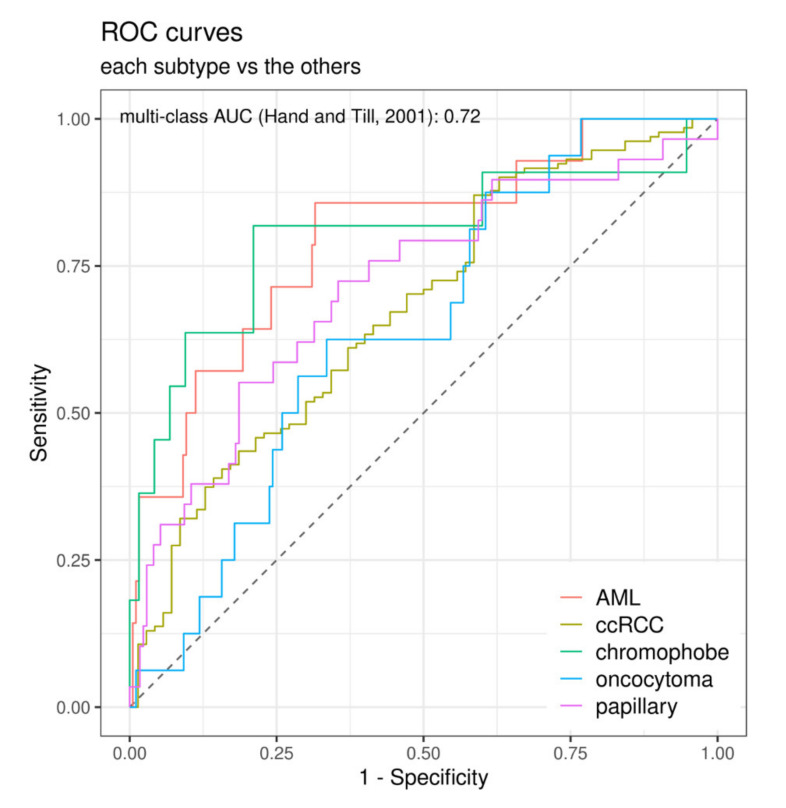
ROC curves for the extreme gradient boosting classifier (XG boost; with SMOTE and without feature selection). The displayed receiver-operating characteristics (ROC) curves show the discrimination of each subtype vs. the remaining ones as determined by the multi-class AUC described by Hand and Till [14]; AML: angiomyolipoma; ccRCC clear cell renal cell carcinoma; AUC: area under the ROC curve.

Figure 3 shows the receiver-operating characteristics (ROC) curves for the best performing setup (xgboost in SMOTE cohort without feature selection) to differentiate the subtypes. In the underlying pairwise comparisons, the highest AUC was achieved for the differentiation of chromophobe RCCs from AMLs (AUC = 0.85). The lowest AUC was evident for the differentiation of oncocytomas from AMLs and chromophobe RCCs (AUC = 0.55 and AUC = 0.45), which stipulated subgroup analyses excluding oncocytomas. All pairwise comparisons are provided in Table 4, while sensitivity and specificity of each machine learning algorithm are summarized in Table S2.

### 2.3. Subgroup Analyses

Based on the lowest AUC for the discrimination of oncocytomas vs. other subtypes in the full cohort, subgroup analyses were conducted in a subpopulation excluding patients with oncocytomas. The baseline characteristics of the subgroup are provided in in Table S3.

In the subpopulation, all machine learning models were retrained in analogy to the analyses in the full cohort. Across all machine learning models, feature selection resulted in diminishing performance, except for k-nearest neighbors (knn) and boosted classification tree (C5.0) (mean AUC decrease 0.05). Upsampling resulted in better classification performance both with feature selection (mean AUC increase 0.05) and without feature selection (mean AUC increase 0.07). The best-performing ML algorithm in this subcohort was a random forest algorithm (ranger) using upsampling that reached a multiclass AUC = 0.78 (Figure S1). The performance of all evaluated ML algorithms is summarized in Table S4.

In order to evaluate the influence of technical heterogeneity in the data, subgroup analyses were conducted on patients with CT scans with slice thickness <3 mm (*n* = 104, Table S5), on external patients only (*n* = 167, Table S6), and on patients without imaging artifacts (*n* = 141, Table S7). The highest diagnostic performance ranged from AUC = 0.68 to AUC = 0.72.

### 2.4. Sensitivity Analyses

For sensitivity analyses, cost sensitivity was introduced by setting class weights to the inverse of the class prevalence in the full study cohort. Given the multi-class approach of our clinical setting and the algorithmic architecture, weighting was not implemented for C5.0 and k-nearest neighbors (knn). There was no relevant AUC increase compared to SMOTE, as demonstrated in Table S8.

## 3. Discussion

The accurate classification of renal tumor subtypes in imaging studies has immediate implications for clinical patient management. Still, using conventional radiological assessment strategies in CT, the discrimination of renal tumor subtypes is imperfect. In particular, incidentally detected renal tumors in CT studies that might not have been specifically tailored for renal tumor evaluation, i.e., monophasic CT studies, pose a radiological challenge.

In this analysis, a large cohort of renal tumor patients who underwent routine venous-phase CT studies was assessed. In a pragmatic approach, patient inclusion was not restricted regarding type of CT scanner, slice thickness, and imaging artifacts to reflect the diversity of CT imaging protocols and quality that is encountered by radiologists in everyday clinical practice. To ensure an accurate reference for further analyses, all renal tumor specimens of the included patients underwent standardized histopathological assessment.

In this setting mimicking a real-world scenario with CT scans from clinical routine CT, radiomic feature analyses and subsequent extensive machine learning algorithms were used to predict the histological subtype of renal tumors preoperatively.

Concerning demographic variables, this study’s population is comparable to those described in the literature, demonstrating a male predominance of renal tumors and mean age of 64 years [5]. The lower frequency of benign renal tumors (15%) compared to that found in the established literature (20–30%) might well be attributable to varying characteristics of the specific subpopulation as well as to a preselection of patients for surgical resection without classic AML imaging features [7,15].

Using different approaches to imbalance handling and feature selection, the best-performing extreme gradient boosting algorithm in the full patient cohort yielded a multiclass AUC = 0.72 for the discrimination of different renal tumor subtypes. On exploratory analyses, it was evident that the discrimination of oncocytomas from other renal tumor subtypes was the most challenging in our dataset. Indeed, in subgroup analyses excluding the oncocytoma subgroup, the ML algorithms performed better than in the full cohort, with a multiclass AUC = 0.78 for a random forest algorithm.

In contrast, the discrimination of oncocytomas from chromophobe RCCs demonstrated the lowest accuracy in our cohort (AUC = 0.45). This might correlate to missing arterial-phase imaging in our study, as Amin et al. reported early contrast enhancement to be most reliable for the discrimination of oncocytomas from chromophobe RCCs [16].

Using synthetic upsampling of minority classes (SMOTE) in our study yielded superior results, which might indicate that upsampling (or weighting) of less common histological subtypes is necessary to obtain robust machine learning algorithms. In this context, it has to be highlighted that upsampling was conducted on the training datasets only, without affecting the validation datasets. Thereby, any erroneous accentuation of specific histological subtypes or imaging features by the upsampling algorithm should have negatively affected the diagnostic performance in the non-upsampled validation dataset.

The overall moderate diagnostic performance of this study might result from the heterogenous imaging data at hand, with 83.1% of the cases acquired at external imaging centers and 29.9% of imaging artifacts, which were mostly observed with higher slice thickness. These heterogenous data with sometimes low imaging quality might well have negatively affected the diagnostic performance of the machine learning algorithms.

Nevertheless, this heterogenous dataset realistically mimics the data and diagnostic challenges modern radiology faces in clinical routine. To further assess the impact of imaging quality on the diagnostic performance of machine learning algorithms, extensive subgroup analyses were conducted. These subgroups were limited to patients with high image quality, namely, those without imaging artifacts and with low slice thickness. Interestingly, the diagnostic performance in all of these scenarios was comparable to that of the full cohort, which might result from a smaller sample size. Conversely, these results indicate that the machine learning algorithms presented in this study robustly perform even in cases with low image quality.

Notably, the diagnostic performance observed in this particular study is inferior to the discrimination between benign and malignant renal tumors (AUC = 0.83) that was reported by our study group for an earlier cohort of 94 patients, which outperformed the assessment by two independent radiologists (AUC = 0.68, *p* =0.047) [17]. These differences might have been driven by the inclusion of renal tumors >7 cm in diameter and a dedicated classification of oncocytomas in the current study, that proved to be most challenging.

In 2017, Coy et al. demonstrated the utility of peak lesion attenuation analyses to discriminate renal tumor histological subtypes with pairwise AUCs ranging between 0.96 and 0.79 [11]. Similar to our results, the diagnostic performance was worse for the discrimination of ccRCC from oncoytomas (AUC = 0.79) and of ccRCC from fat-poor AMLs (AUC = 0.83), indicating that these subtypes have overlapping imaging features. One explanation for the overall higher diagnostic performance of Coy et al. might be the utilization of computer-aided renal tumor segmentation and imaging during the corticomedullary contrast-media phase obtained from standardized CT protocols at one institution. In contrast, our patient cohort was imaged in the more readily available venous phase and manually segmented. CT studies included in our work were purposely obtained from multiple imaging centers with various CT scanners, including studies with large slice thickness and imaging artifacts. Therefore, our results might provide a more realistic estimate on how well radiomic feature analyses and ML algorithms perform in a real-world scenario in a heterogeneous patient population.

There are several published studies regarding machine learning in renal imaging [18,19,20,21]. Notably, using support vector machines (SVMs) in a cohort of *n* = 58 patients, Feng et al. assessed their diagnostic potential to discriminate between RCCs and fat-poor AMLs, with an accuracy of nearly 94% [12]. Kocak et al. evaluated *n* = 68 patients and described an accuracy for discrimination of non-clear-cell RCC from ccRCC of 85%, while the accuracy for renal tumor subtype assessment was lower, at 69% [13]. Yu et el. evaluated renal tumors in *n* = 119 patients using radiomic features and SVM and reported pairwise comparisons between selected renal tumor subtypes with AUC up to 92%, although a joint model for comprehensive assessment of all renal tumor subtypes was not reported [10].

Although these studies reported promising results, they were mostly designed to only include CT studies from selected imaging centers, often with standardized multiphasic protocols. As demonstrated in our study, renal CT imaging in clinical reality varies regarding imaging quality, available slice thickness, and acquisition protocol, with several patients presenting with imaging studies from external imaging centers. Therefore, the question remains of how well the promising results from standardized imaging studies are generalizable to a broader clinical population.

The underlying biopathological changes associated with specific radiomic feature profiles of renal tumor subtypes are not fully understood yet. Some authors hypothesized that radiomic features correlate with the degree of microvessel density [22] and changes in renal cell metabolism [23,24,25].

This study has several limitations: first, given the geographical location of the tertiary center where renal tumor resections were performed, only patients of Caucasian race from a restricted European region were evaluated, which could limit the generalizability of the results to a more diverse population. Second, given the sample size of the evaluated patient cohort, no independent validation dataset was created, and results of the machine learning algorithms were based on cross-validation, which might have resulted in overfitted and overly optimistic results. Third, restriction of analyses to the most common renal tumor subtypes ccRCC, papillary and chromophobe RCC, AML, and oncocytoma does not reflect the clinical diversity of renal neoplasms and might thus limit the applicability of the presented results to specific cases. Further, the inclusion of patients receiving surgical resection resulted in the exclusion of those with classical imaging presentation of fat-rich AMLs who were correctly identified in CT or MRI studies. Thus, the presented algorithms might yield differing diagnostic performance in cohorts without preselection. Moreover, no substratification of type 1 and type 2 papillary RCC was conducted in this study, given the small sample size and the large number of histological subtypes already evaluated. Finally, the lack of an independent, external validation dataset might limit the generalizability of our results, although extensive measures, such as cross-validation, were applied in our study. Given this limitation, our algorithms might have been overfitted, and the generalizability of our findings to other patient populations might be poor.

The algorithm presented in this study has several potential clinical applications: these include patient stratification for immediate treatment or watchful waiting, as well as identification of patients who might benefit from additional imaging studies, such as multiphasic CT studies or magnetic resonance imaging. Further, the algorithm could aid in finding consensus in cases of discordant renal tumor assessment by two radiologists and provide a comprehensive decision support to tumor boards.

## 4. Material and Methods

This retrospective study received prior approval by the ethics committee of the University Medical Center Goettingen (No 2/4/17, approved 24 March 2017) and is compliant with the Declaration of Helsinki. Of the patients included in this study, *n* = 94 were previously described in analyses that evaluated the diagnostic performance of radiomic features and machine learning to discriminate between malignant and benign renal tumors [17]. This study added *n* = 107 patients and focused on histological subtype assessment.

### 4.1. Study Cohort Selection

Adult patients with renal tumors consecutively presenting for surgical resection between 2012 and 2019 at the University Medical Center Goettingen who had received preoperative, contrast-enhanced CT imaging in venous phase, were included in this study. Analyses were restricted to patients diagnosed with clear cell, papillary, or chromophobe RCC or AML, irrespective of tumor diameter.

Patients with diffusely infiltrative tumors (i.e., lymphoma or chronic inflammatory changes) as well as those presenting with cystic neoplasms or Bosniak IV cysts were excluded, due to concerns regarding segmentation validity.

### 4.2. CT Imaging

In a pragmatic study design, CT studies from multiple outside imaging centers and our tertiary referral center were included. CT studies were included if they were performed after administration of intravenous iodinated contrast media, acquired in venous phase, depicting the complete kidneys, and provided as axial reconstructions using a soft-tissue kernel. For the scope of this study, venous-phase imaging was defined as homogenous enhancement of the renal parenchyma. No restrictions were made regarding CT scanner type, specific acquisition protocols or reconstructions, slice thickness, or potential imaging artifacts. Imaging artifacts were categorized on a subjective Likert scale as mild, moderate, and severe.

### 4.3. Radiomic Feature Analyses

Manual renal tumor segmentation and radiomic feature analyses were performed using the open source software “3D Slicer” and the PyRadiomics plugin by a trained reader delineating the region of interest (ROI) on axial slices [26,27]. The radiomic feature subtypes evaluated in this study are detailed in Table S9, following feature definitions by the Imaging Biomarker Standardization Initiative to ensure a standardized and reproducible approach [27,28]. All radiomic feature analyses were performed using a bin width of 25. All radiomic features were one-dimensional for downstream use in machine learning models.

### 4.4. Renal Tumor Assessment

As a gold standard, all renal tumors (partial or radical nephrectomy specimens) were histopathologically assessed at the Department of Pathology, University Medical Center Goettingen, using hematoxylin–eosin staining, as well as immunostaining for cytokeratin 7, cluster of differentiation (CD) 10 and CD117, as well as vimentin. For the establishment of AML diagnosis, staining for Melan-A, human melanoma black (HMB) 45, and actin was performed.

### 4.5. Machine Learning

Radiomic features with near-zero variance were discarded from the analysis, and only one representative of variables with identical information was retained. The remaining data were screened for linear dependencies which were broken by discarding one of the columns in the linear dependent groups. The filtered dataset consisted of 127 radiomics features next to age and gender.

Internal validation using resampling was applied in order to assess the generalization performance of the applied methodology. We used a 10-fold cross-validation (CV) scheme leading to 9 folds with test sets of size 20 and 1 fold with a test set of size 21. Stratified sampling was applied to approximate the subtype distribution of renal tumors in all training and test sets. All further steps were conducted within each fold of each repetition of the CV to avoid information leakage.

Two strategies to handle the class imbalance in the data were pursued: (1) no special handling of class imbalance and (2) SMOTE, which oversamples the minority classes using the nearest neighbors of the cases in these classes [29]. SMOTE was applied pairwise for each of the minority classes in comparison with the remaining samples. The amount of oversampling was set to achieve approximately half the number of samples in the majority class. As a sensitivity analysis (cost sensitivity), class imbalance was addressed by setting sample weights to the inverse prevalence of their class.

Three strategies for feature selection were pursued: (1) no feature selection, i.e., all features were passed to the ML models; (2) recursive feature elimination (RFE), i.e., a random forest model was fit to the data, and the features were ordered by importance in that classifier. Then, for each *n*, a classifier was trained using the top *n* features only, and the features yielding the best performance were retained. An internal 10-fold cross-validation was used in order to avoid bias [30]; (3) feature extraction via PCA. Within each fold, a PCA was conducted on the training set, and the principle components cumulative explaining 80% of the variance were retained. The test samples were projected onto the same PCA.

Using the two strategies to handle class imbalance and the radiomics features from the two strategies to select features, the following 8 machine learning algorithms were trained (further details and tuning parameters provided in Table S10): random forest (RF), random forest ranger (ranger), extreme gradient boosting (XG boost), boosted classification trees (C5.0), elastic-net penalized multinomial regression (glmnet), support vector machine (SVM), k-nearest neighbor (Knn), and neural network (NN).

### 4.6. Statistical Analyses and Diagnostic Performance Assessment

The diagnostic performance of the ML algorithms was assessed using the AUC in a generalization to multi-class problems proposed by Hand and Till, 2001, which aggregates the AUC over all pairwise comparisons [14]. All reported diagnostic performance measures were derived from out-of-bag samples of the 10-fold cross-validation. Sensitivity and specificity in the classification of each subtype vs. the others were calculated using a fixed cutoff at 0.5.

All statistical analyses were performed using R and RStudio with the R package “caret” [31]. An alpha level of 0.05 was chosen to indicate statistical significance. All provided *p*-values are two-sided.

## 5. Conclusions

In a large, heterogenous patient cohort with venous-phase CTs from different imaging centers, radiomic feature analyses and machine learning algorithms predicted renal tumor subtypes with moderate accuracy. The discrimination of oncocytomas from other renal tumor subtypes proved to be the most challenging.

This pragmatic approach might aid in clinical patient management, stratifying patients for immediate treatment, watchful waiting, or additional imaging, including multiphasic CT studies or magnetic resonance imaging.

## Figures and Tables

**Table 1:** Differences in patient age, gender, and largest three-dimensional (3D) diameter according to renal tumor subtype in the full cohort without and with the synthetic minority oversampling technique (SMOTE): ccRCC, clear cell renal cell carcinomas, AML, angiomyolipomas.

Cohort	Parameter	Level	Total	ccRCC	Papillary	Oncocytoma	AML	Chromophobe	*p* Value
Full cohort without SMOTE	*n*		201	131	29	16	14	11	
	age	mean ± sd	64 ± 11	65 ± 11	67 ± 11	63 ± 8.4	56 ± 14	60 ± 9.2	0.02
		median (min; max)	66 (31; 85)	68 (31; 85)	68 (40; 81)	62 (51; 77)	56 (31; 76)	59 (47; 78)	
	gender	female	73 (36.3%)	43 (32.8%)	5 (17.2%)	9 (56.2%)	9 (64.3%)	7 (63.6%)	<0.01
		male	128 (63.7%)	88 (67.2%)	24 (82.8%)	7 (43.8%)	5 (35.7%)	4 (36.4%)	
	Max 3D Diameter	mean ± sd	58 ± 28	57 ± 23	57 ± 35	58 ± 30	51 ± 35	72 ± 48	0.46
		median (min; max)	52 (13; 192)	54 (21; 144)	47 (22; 183)	52 (20; 141)	43 (13; 127)	59 (16; 192)	
Full cohort with SMOTE	*n*		389	131	58	64	70	66	
	age	mean ± sd	62 ± 11	65 ± 11	67 ± 9.7	64 ± 6.6	55 ± 11	59 ± 7.2	<0.01
		median (min; max)	63 (31; 85)	68 (31; 85)	69 (40; 81)	65 (51; 77)	56 (31; 76)	59 (47; 78)	
	gender	female	176 (45.2%)	43 (32.8%)	9 (15.5%)	38 (59.4%)	43 (61.4%)	43 (65.2%)	<0.01
		male	213 (54.8%)	88 (67.2%)	49 (84.5%)	26 (40.6%)	27 (38.6%)	23 (34.8%)	
	Max. 3D Diameter	mean ± sd	57 ± 28	57 ± 23	56 ± 30	54 ± 24	51 ± 29	70 ± 35	<0.01
		median (min; max)	51 (13; 192)	54 (21; 144)	47 (22; 183)	50 (20; 141)	46 (13; 127)	64 (16; 192)	

*n*: number; sd: standard deviation; ccRCC: clear cell renal cell carcinoma; AML: angiomyolipoma.

**Table 2:** Summary of imaging artifacts, CT slice thickness, and imaging center. IQR, inter-quartile range.

Parameter	Level	Total	Any Imaging Artifacts	No Imaging Artifacts	*p* Value
*n*		201	60	141	
imaging center	external imaging center	167 (83.1%)	58 (96.7%)	109 (77.3%)	<0.01
	tertiary imaging center	34 (16.9%)	2 (3.3%)	32 (22.7%)	
slice thickness	mean ± sd		2.79 ± 1.81	4.3 ± 1.31	<0.01
	median (IQR)	2 (1–5)	5 (4.5–5)	1.2 (1–3)	

*n*: number; sd: standard deviation; IQR: inter-quartile-range.

**Table 3:** Area under the receiver-operating characteristics (ROC) curve (AUC) for the combinations of various machine learning models, feature selection, and imbalance handling.

	No Upsampling			SMOTE		
Machine Learning Algorithm	No Feature Selection	RFE	PCA	No Feature Selection	RFE	PCA
C5.0	0.65	0.58	0.65	0.71	0.65	0.63
glmnet	0.64	0.66	0.65	0.66	0.69	0.68
knn	0.58	0.57	0.59	0.59	0.58	0.56
nnet	0.63	0.57	0.60	0.68	0.67	0.64
ranger	0.68	0.63	0.70	0.69	0.63	0.72
rf	0.68	0.65	0.67	0.7	0.64	0.70
svmRadial	0.65	0.58	0.66	0.65	0.62	0.67
xgboost	0.7	0.62	0.67	0.72	0.67	0.71

C5.0: boosted classification tree; glmnet: elastic-net penalized multinomial regression; knn: k-nearest neighbors; nnet: neural network; rf: random forest; svmRadial: support vector machine using a radial kernel; xgboost: extreme gradient boosting; ranger: random forest variant; RFE: recursive feature elimination; PCA: principle component analysis; SMOTE: synthetic minority oversampling technique.

**Table 4:** Pairwise discrimination performance of the extreme gradient boosting algorithm in the full cohort with SMOTE and without feature selection. The multi-class AUC is based on averaging AUCs from all pairwise subtype comparisons. This table shows the mean AUC from these pairwise comparisons.

Pair	AUC
ccRCC/AML	0.77
ccRCC/chromophobe	0.71
ccRCC/oncocytoma	0.67
ccRCC/papillary	0.67
papillary/AML	0.75
papillary/chromophobe	0.62
papillary/oncocytoma	0.74
oncocytoma/AML	0.55
oncocytoma/chromophobe	0.45
AML/chromophobe	0.85

AML: angiomyolipoma; ccRCC clear cell renal cell carcinoma; AUC: area under the receiver-operating characteristics curve.

## Supplementary Materials

The following are available online at https://www.mdpi.com/2072-6694/12/10/3010/s1, Figure S1: ROC curves for the random forest “ranger” classifier in the upsampled subcohort excluding oncocytomas, with SMOTE and without feature selection, Table S1: details of histological grading and T stage of malignant renal tumor subtypes, Table S2. Sensitivity/specificity in the classification of different subtypes for the combinations of various machine learning models, feature selection, and imbalance handling methods, Table S3: Differences in patient age, gender, and largest 3-dimensional (3D) diameter according to renal tumor subtype in the cohort excluding oncocytomas, without and with synthetic minority oversampling technique (SMOTE), Table S4: Area under the receiver-operating characteristics curve (AUC) for the combinations of various machine learning models, feature selection, and imbalance handling in the subgroup analysis excluding patients with oncocytomas, Table S5: Area under the receiver-operating characteristics curve (AUC) for the combinations of various machine learning models, feature selection, and imbalance handling in the subgroup analysis including only the *n* = 104 patients with CT scans with slice thickness <3 mm, Table S6: Area under the receiver-operating characteristics curve (AUC) for the combinations of various machine learning models, feature selection, and imbalance handling in the subgroup analysis including only the *n* = 167 external patients, Table S7: Area under the receiver-operating characteristics curve (AUC) for the combinations of various machine learning models, feature selection, and imbalance handling in the subgroup analysis including only the *n* = 141 patients without artefacts in the CT scans, Table S8: AUC for the combinations of various machine learning models and feature selection with class weights set as inverse class probability, Table S9: Details of the evaluated radiomic features, Table S10: Details of the evaluated machine learning algorithms.

## Abbreviations

AML	angiomyolipoma
AUC	area under the ROC curve
CD	cluster of differentiation
CK	cytokeratin
ccRCC	clear cell renal cell carcinoma
CT	computed tomography
C5.0	boosted classification tree
glmnet	elastic net penalized multinomial regression
HE	hematoxylin-eosin
HMB	human melanoma black
ICC	interobserver correlation coefficient
IQR	inter-quartile range
KNN	k-nearest neighbor
nnet	neural network
POM	probability of malignancy
RCC	renal cell carcinoma
RF	random forest
RFE	recursive feature elimination
ROC	receiver operating characteristics
ROI	region of interest
SMOTE	synthetic minority oversampling technique
SVM	support vector machine
US	United States
xgboost	extreme gradient boosting
