# Supplementary material for: Radiomic Features and Machine Learning for the Discrimination of Renal Tumor Histological Subtypes: A Pragmatic Study Using Clinical-Routine Computed Tomography
Source: Cancers (Basel). 2020 Oct 16;12(10):3010. doi: 10.3390/cancers12103010 (PMC7603020; doi:10.3390/cancers12103010)
Supplement: Supplementary file 1 [file cancers-12-03010-s001.pdf]

Supplementary Material: Radiomic Features and Machine Learning for Discrimination of Renal Tumor Histological Subtypes: A Pragmatic Study Using Clinical Routine Computed Tomography

Johannes Uhlig, Andreas Leha, Laura M. Delonge, Anna-Maria Haack, Brian Such, Hyun S. Kim, Felix Bremmer, Lutz Trojan, Joachim Lotz, Annemarie Uhlig

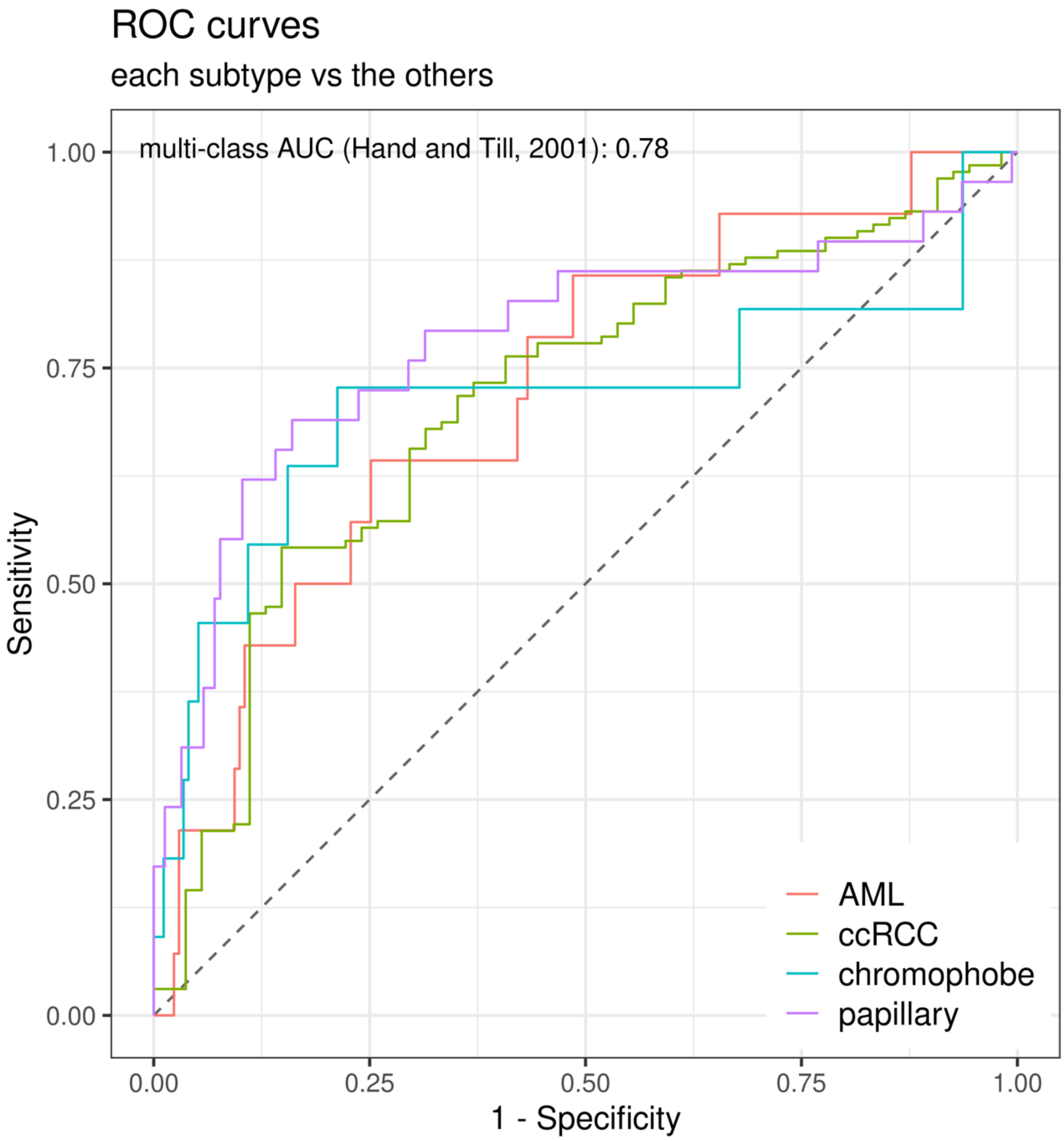

Figure S1. ROC curves for the random forest "ranger" classifier in the upsampled subcohort excluding oncocytomas, with SMOTE and without feature selection. The displayed receiver-operating characteristics (ROC) curves show the discrimination of each subtype vs. the remaining ones as determined by the multi-class AUC described by Hand and Till [30]. AML: angiomyolipoma; ccRCC clear cell renal cell carcinoma; AUC: area under the ROC curve.

Table S1. Details on histological grading and T stage of malignant renal tumor subtypes.

Parameter	Level	Total	ccRCC	Chromophobe	Papillary	*p* Value
n		171	131	11	29	
histological grading						0.45
	G1	27 (15.8%)	20 (15.3%)	1 (9.1%)	6 (20.7%)	
	G2	118 (69.0%)	91 (69.5%)	7 (63.6%)	20 (69.0%)	
	G3	7 (4.1%)	7 (5.3%)	0 (0.0%)	0 (0.0%)	
	Gx	19 (11.1%)	13 (9.9%)	3 (27.3%)	3 (10.3%)	
T stage						0.55
	T1	137 (80.1%)	107 (81.7%)	8 (72.7%)	22 (75.9%)	
	T2	8 (4.7%)	4 (3.1%)	2 (18.2%)	2 (6.9%)	
	T3	23 (13.5%)	17 (13.0%)	1 (9.1%)	5 (17.2%)	
	T4	1 (0.6%)	1 (0.8%)	0 (0.0%)	0 (0.0%)	
	Tx	2 (1.2%)	2 (1.5%)	0 (0.0%)	0 (0.0%)	

n: number; ccRCC: clear cell renal cell carcinoma.

Table S2. Sensitivity/specificity in the classification of different subtypes for the combinations of various machine learning models, feature selection and imbalance handling methods.

Machine Learning Algorithm	Subtype	No Upsampling			SMOTE		
		No FS	RFE	PCA	No FS	RFE	PCA
C5.0	AML	0.20/0.98	0.35/0.97	0.10/0.98	0.15/0.96	0.35/0.93	0.10/0.94
	ccRCC	0.88/0.29	0.87/0.23	0.87/0.24	0.76/0.31	0.79/0.47	0.69/0.43
	chromophobe	0.20/0.96	0.20/0.96	0.10/0.97	0.20/0.95	0.10/0.98	0.20/0.91
	oncocytoma	0.05/0.98	0.00/0.99	0.05/0.98	0.00/0.94	0.00/0.91	0.10/0.94
	papillary	0.23/0.94	0.07/0.95	0.17/0.93	0.30/0.93	0.33/0.93	0.25/0.91
glmnet	AML	0.15/0.97	0.40/0.97	0.25/0.99	0.45/0.93	0.55/0.95	0.30/0.95
	ccRCC	0.92/0.23	0.93/0.24	0.96/0.13	0.67/0.56	0.75/0.54	0.78/0.41
	chromophobe	0.10/0.98	0.10/0.98	0.10/0.98	0.20/0.95	0.20/0.94	0.35/0.92
	oncocytoma	0.00/0.99	0.00/0.99	0.00/1.00	0.10/0.88	0.05/0.90	0.05/0.96
	papillary	0.08/0.94	0.12/0.96	0.03/0.98	0.28/0.89	0.32/0.93	0.17/0.94
knn	AML	0.00/1.00	0.00/0.99	0.05/0.99	0.15/0.91	0.20/0.93	0.25/0.94
	ccRCC	0.99/0.03	0.98/0.04	0.94/0.07	0.49/0.76	0.52/0.69	0.60/0.67
	chromophobe	0.00/1.00	0.00/1.00	0.10/0.99	0.25/0.87	0.25/0.88	0.20/0.87
	oncocytoma	0.00/1.00	0.00/0.99	0.00/1.00	0.20/0.79	0.20/0.81	0.25/0.87
	papillary	0.00/0.98	0.00/0.98	0.03/0.95	0.32/0.87	0.18/0.86	0.20/0.87
nnet	AML	0.15/0.96	0.15/0.93	0.20/0.98	0.50/0.91	0.50/0.88	0.55/0.90
	ccRCC	0.73/0.53	0.95/0.20	0.90/0.24	0.61/0.56	0.80/0.40	0.81/0.37
	chromophobe	0.00/0.99	0.00/1.00	0.00/0.98	0.30/0.92	0.25/0.95	0.05/0.96
	oncocytoma	0.05/0.97	0.00/1.00	0.10/0.99	0.15/0.85	0.00/0.97	0.05/0.97
	papillary	0.37/0.74	0.07/0.98	0.10/0.92	0.20/0.92	0.03/0.95	0.07/0.96
ranger	AML	0.10/0.99	0.00/0.99	0.15/0.99	0.15/0.97	0.20/0.97	0.10/0.95
	ccRCC	0.95/0.09	0.96/0.04	0.94/0.13	0.86/0.21	0.92/0.17	0.78/0.30
	chromophobe	0.00/0.99	0.00/0.99	0.00/0.98	0.25/0.94	0.10/0.97	0.10/0.92
	oncocytoma	0.00/0.99	0.00/1.00	0.00/0.99	0.00/0.98	0.00/0.99	0.15/0.97
	papillary	0.07/0.98	0.03/0.98	0.15/0.97	0.07/0.97	0.07/0.98	0.13/0.95
rf	AML	0.00/0.99	0.00/0.99	0.15/0.99	0.10/0.96	0.40/0.97	0.15/0.92
	ccRCC	0.95/0.06	0.96/0.07	0.92/0.14	0.91/0.26	0.89/0.23	0.79/0.34
	chromophobe	0.00/0.98	0.00/0.99	0.10/0.98	0.25/0.94	0.20/0.97	0.30/0.92
	oncocytoma	0.00/0.99	0.05/0.99	0.00/0.99	0.00/0.99	0.00/0.99	0.00/0.97
	papillary	0.03/0.98	0.03/0.98	0.15/0.96	0.12/0.98	0.07/0.96	0.22/0.97
svmRadial	AML	0.00/1.00	0.00/1.00	0.00/1.00	0.00/0.99	0.10/1.00	0.00/0.99
	ccRCC	0.98/0.01	0.98/0.00	0.99/0.01	0.88/0.27	0.89/0.30	0.85/0.17
	chromophobe	0.00/0.99	0.00/0.99	0.00/0.99	0.15/0.97	0.15/0.98	0.00/0.96
	oncocytoma	0.00/1.00	0.00/1.00	0.00/1.00	0.00/1.00	0.00/0.99	0.00/0.98
	papillary	0.00/0.98	0.00/0.99	0.00/0.99	0.28/0.89	0.35/0.90	0.18/0.92
xgbTree	AML	0.05/0.99	0.20/0.98	0.15/0.99	0.25/0.95	0.55/0.94	0.15/0.93
	ccRCC	0.90/0.26	0.92/0.26	0.91/0.20	0.78/0.41	0.80/0.41	0.74/0.39
	chromophobe	0.20/0.98	0.10/0.98	0.10/0.97	0.10/0.97	0.05/0.97	0.05/0.95
	oncocytoma	0.00/0.98	0.05/0.98	0.00/0.99	0.05/0.96	0.05/0.95	0.25/0.96

	papillary	0.22/0.92	0.07/0.94	0.13/0.95	0.35/0.89	0.28/0.93	0.28/0.90

FS: feature selection; glmnet: elastic net penalized multinomial regression; nnet: neural network; rf: random forest; svmRadial: support vector machine using a radial kernel; xgboost: extreme gradient boosting; RFE: recursive feature elimination; PCA: principle component analysis.

Table S3. Differences in patient age, gender, and largest 3-dimensional (3D) diameter according to renal tumor subtype in the cohort excluding oncocytomas, without and with synthetic minority oversampling technique (SMOTE).

Cohort	Parameter	Level	Total	ccRCC	Papillary	AML	Chromophobe	*p* Value
Without SMOTE	n		185	131	29	14	11	
	age							0.01
		mean ± sd	65 ± 12	65 ± 11	67 ± 11	56 ± 14	60 ± 9.2	
		median (min; max)	66 (31; 85)	68 (31; 85)	68 (40; 81)	56 (31; 76)	59 (47; 78)	
	gender							<0.01
		female	64 (34.6%)	43 (32.8%)	5 (17.2%)	9 (64.3%)	7 (63.6%)	
		male	121 (65.4%)	88 (67.2%)	24 (82.8%)	5 (35.7%)	4 (36.4%)	
	Maximum3DDiameter							0.30
		mean ± sd	58 ± 28	57 ± 23	57 ± 35	51 ± 35	72 ± 48	
		median (min; max)	52 (13; 192)	54 (21; 144)	47 (22; 183)	43 (13; 127)	59 (16; 192)	
With SMOTE	n		325	131	58	70	66	
	age							<0.01
		mean ± sd	62 ± 11	65 ± 11	66 ± 11	57 ± 12	59 ± 7.7	
		median (min; max)	64 (31; 85)	68 (31; 85)	68 (40; 81)	56 (31; 76)	59 (47; 78)	
	gender							<0.01
		female	146 (44.9%)	43 (32.8%)	8 (13.8%)	47 (67.1%)	48 (72.7%)	
		male	179 (55.1%)	88 (67.2%)	50 (86.2%)	23 (32.9%)	18 (27.3%)	
	Maximum3DDiameter							<0.01
		mean ± sd	57 ± 28	57 ± 23	55 ± 31	48 ± 27	68 ± 32	
		median (min; max)	51 (13; 192)	54 (21; 144)	46 (22; 183)	41 (13; 127)	62 (16; 192)	

n: number; sd: standard deviation; ccRCC: clear cell renal cell carcinoma; AML: angiomyolipoma.

Table S4. Area under the receiver-operating characteristics curve (AUC) for the combinations of various machine learning models, feature selection and imbalance handling in the subgroup analysis excluding patients with oncocytomas.

	No Upsampling			SMOTE		
**Machine Learning Algorithm**	**No Feature Selection**	**RFE**	**PCA**	**No Feature Selection**	**RFE**	**PCA**
C5.0	0.59	0.65	0.60	0.74	0.70	0.73
glmnet	0.67	0.67	0.68	0.69	0.68	0.71
knn	0.57	0.63	0.59	0.62	0.64	0.62
nnet	0.65	0.63	0.66	0.68	0.67	0.73
ranger	0.73	0.64	0.74	0.78	0.65	0.75
rf	0.70	0.64	0.75	0.75	0.65	0.73
svmRadial	0.71	0.63	0.72	0.72	0.64	0.71
xgboost	0.70	0.64	0.69	0.73	0.66	0.72

C5.0: boosted classification tree; glmnet: elastic net penalized multinomial regression; knn: k-nearest neighbors; nnet: neural network; rf: random forest; svmRadial: support vector machine using a radial kernel; xgboost: extreme gradient boosting; rfe: recursive feature elimination; PCA: principle component analysis; SMOTE: synthetic minority oversampling technique.

Table S5. Area under the receiver-operating characteristics curve (AUC) for the combinations of various machine learning models, feature selection and imbalance handling in the subgroup analysis including only the *n* = 104 patients with CT scans with slice thickness <3mm.

	No Upsampling			SMOTE		
**Machine Learning Algorithm**	**No FS**	**RFE**	**PCA**	**No FS**	**RFE**	**PCA**
C5.0	0.62	0.63	0.59	0.57	0.62	0.64
glmnet	0.56	0.62	0.64	0.60	0.64	0.70
knn	0.57	0.55	0.61	0.58	0.54	0.59
nnet	0.61	0.60	0.61	0.60	0.54	0.60
ranger	0.67	0.58	0.68	0.66	0.59	0.68
rf	0.69	0.58	0.68	0.66	0.57	0.70
svmRadial	0.69	0.63	0.67	0.66	0.63	0.64
xgboost	0.68	0.66	0.63	0.64	0.64	0.72

C5.0: boosted classification tree; glmnet: elastic net penalized multinomial regression; knn: k-nearest neighbors; nnet: neural network; rf: random forest; svmRadial: support vector machine using a radial kernel; xgboost: extreme gradient boosting; FS: feature selection; RFE: recursive feature elimination; PCA: principle component analysis; SMOTE: synthetic minority oversampling technique.

Table S6. Area under the receiver-operating characteristics curve (AUC) for the combinations of various machine learning models, feature selection and imbalance handling in the subgroup analysis including only the *n* = 167 external patients.

	No Upsampling			SMOTE		
**Machine Learning Algorithm**	**No FS**	**RFE**	**PCA**	**No FS**	**RFE**	**PCA**
C5.0	0.47	0.40	0.56	0.56	0.47	0.63
glmnet	0.64	0.64	0.64	0.68	0.68	0.62
knn	0.38	0.37	0.39	0.41	0.40	0.40
nnet	0.55	0.46	0.54	0.64	0.60	0.62
ranger	0.64	0.60	0.68	0.64	0.60	0.66
rf	0.62	0.60	0.64	0.66	0.64	0.64
svmRadial	0.63	0.62	0.64	0.65	0.60	0.64
xgboost	0.68	0.63	0.60	0.64	0.62	0.63

C5.0: boosted classification tree; glmnet: elastic net penalized multinomial regression; knn: k-nearest neighbors; nnet: neural network; rf: random forest; svmRadial: support vector machine using a radial kernel; xgboost: extreme gradient boosting; FS: feature selection; RFE: recursive feature elimination; PCA: principle component analysis; SMOTE: synthetic minority oversampling technique.

Table S7. Area under the receiver-operating characteristics curve (AUC) for the combinations of various machine learning models, feature selection and imbalance handling in the subgroup analysis including only the *n* = 141 patients without artefacts in the CT scans.

	No Upsampling			SMOTE		
**Machine Learning Algorithm**	**No FS**	**RFE**	**PCA**	**No FS**	**RFE**	**PCA**
C5.0	0.56	0.55	0.55	0.66	0.62	0.57
glmnet	0.68	0.61	0.65	0.68	0.61	0.67
knn	0.57	0.57	0.60	0.62	0.53	0.64
nnet	0.61	0.60	0.57	0.69	0.59	0.59
ranger	0.62	0.59	0.69	0.64	0.59	0.69
rf	0.66	0.58	0.65	0.66	0.58	0.66
svmRadial	0.64	0.63	0.70	0.64	0.60	0.68
xgboost	0.68	0.61	0.63	0.68	0.61	0.64

C5.0: boosted classification tree; glmnet: elastic net penalized multinomial regression; knn: k-nearest neighbors; nnet: neural network; rf: random forest; svmRadial: support vector machine using a radial kernel; xgboost: extreme gradient boosting; FS: feature selection; RFE: recursive feature elimination; PCA: principle component analysis; SMOTE: synthetic minority oversampling technique.

Table S8. Area under the receiver-operating characteristics curve (AUC) for the combinations of various machine learning models and feature selection with class weights set as inverse class probability.

Machine Learning Algorithm	Weighting Without Feature Selection	Weighting With RFE	Weighting With PCA
glmnet	0.67	0.66	0.68
nnet	0.65	0.65	0.6
ranger	0.69	0.72	0.64
rf	0.7	0.69	0.65
svmRadial	0.33	0.32	0.37
xgboost	0.5	0.5	0.5

glmnet: elastic net penalized multinomial regression; nnet: neural network; rf: random forest; svmRadial: support vector machine using a radial kernel; xgboost: extreme gradient boosting; rfe: recursive feature elimination; PCA: principle component analysis.

Table S9. Details on evaluated radiomic features.

Radiomic Feature Class	Abbreviation	Summary
first-order statistic		describing renal mass voxel intensity
3D shape features		describing 3-dimensional size and shape of renal mass
2D shape features		describing 2-dimensional size and shape of renal mass
gray level co-occurrence matrix features	GLCM	describing second-order joint probability function of renal mass
gray level size zone matrix features	GLSZM	quantifying gray level zones in renal mass
gray level run length matrix features	GLRLM	quantifying gray level runs in renal mass
neighboring gray tone difference matrix features	NGTDM	quantifying the difference between a gray value and average gray value of its neighbors in renal mass
gray level dependence matrix features	GLDM	quantifying gray level dependencies of renal mass

Table S10. Details on evaluated machine learning algorithms.

Radiomic Feature Class	Abbreviation	Summary
Random forest	RF	used 1000 bootstrap samples and majority votes to reach a final model. At each tree node, a random subset of size mtry of variables from the full sample was provided, of which the algorithm picks the one best classifying the respective outcome. The size of the variable subset at each node as well as the size of the terminal node up unto which the tree is grown were optimized using the out-of-bag (OOB) error in a grid search.
Random Forest	ranger	a different implementation of random forests. Single trees were fit to their full size (min.node.size = 1). At each tree node, a random subset of size mtry of variables from the full sample was provided, of which the algorithm picks the one best classifying the respective outcome. The samples are split into two groups according to this variable. The splitting rule (splitrule) as well as the number of randomly selected variables (mtry) were optimized in an internal CV.
Extreme gradient boosting	XG boost	tree-based optimization and boosting model. The number of boosting iterations (nrounds) was set to 100 with early stopping allowed after 3 iterations without performance improvement. The maximum tree depth (max_depth), the shrinkage (eta) to control learning rate, and the minimum sum of instance weight (min_child_weight) to indicate the minimum number of observations in a terminal node were optimized using an internal CV.
Boosted classification trees	C5.0	AdaBoost type boosting of C5.0 decision trees. The number of boosting iterations (trials), whether to use trees or rule-based classifiers (model) and whether to enable winnowing (winnow) have been optimized in an internal CV.
Elastic net penalized	glmnet	The parameter to scale between lasso and ridge regression (alpha) and the penalization factor (lambda) have been optimized in an internal CV.

multinomial regression		
Support vector machine	SVM	creates a multidimensional space with one dimension for each input variable, using a radial kernel. A hyperplane is fit for optimized discrimination of plotted observations which maximizes the distance between outcome classes (margin). Tuning parameters are the degree of self-regularization (cost) and a kernel-specific parameter to define the gaussian distribution (sigma). These were optimized using an internal CV.
k-nearest neighbors	KNN	plots observations in multidimensional space and compares each observation to its neighbors with regard to covariates and outcomes. Tuning parameter is the number of neighbors (k), of which one given observation is compared to for prediction of its outcome.
Neural network	NN	The NN architecture contains one input layer, one hidden layer, and one output layer that are interconnected with links and associated weights. Tuning parameters include the number of nodes in the hidden layer (size), and the weight decay (decay) that penalizes highly influential weights to regulate the network.

Publisher's Note: MDPI stays neutral with regard to jurisdictional claims in published maps and institutional affiliations.

© 2020 by the authors. Licensee MDPI, Basel, Switzerland. This article is an open access article distributed under the terms and conditions of the Creative Commons Attribution (CC BY) license (http://creativecommons.org/licenses/by/4.0/).
